# Lymphoepithelioma-Like Carcinoma of the Urinary Bladder: A Case Report and Review of the Literature

**DOI:** 10.7759/cureus.21281

**Published:** 2022-01-16

**Authors:** Iraklis Mitsogiannis, Lazaros Tzelves, Maria Mitsogianni, Stephanie Vgenopoulou

**Affiliations:** 1 2nd Department of Urology, Sismanoglio General Hospital, National and Kapodistrian University of Athens, Athens, GRC; 2 Department of Pathology, Sismanoglio General Hospital, Athens, GRC

**Keywords:** cisplatin, transurethral resection, urinary bladder carcinoma, epstein-barr virus, urinary bladder, lymphoepithelioma-like carcinoma

## Abstract

Lymphoepithelioma-like carcinoma of the urinary bladder is a rare variant of infiltrating urothelial carcinoma. Diagnostic and therapeutic manipulations are not yet standardised, due to the rarity of the tumour, with surgery and chemotherapy being reported as potential therapeutic options. We report on a case of lymphoepithelioma-like carcinoma of the bladder in a 93-year-old female patient and discuss the pathological features and therapeutic options of the neoplasm. Due to her increased age and associated comorbidities such as hypertension, diabetes mellitus and ischemic heart disease, the patient was treated with transurethral resection of the tumour and subsequent cisplatin-based chemotherapy but unfortunately died of chemotherapy-related complications.

## Introduction

Lymphoepithelioma-like carcinoma (LELC) is an undifferentiated carcinoma of the nasopharynx, commonly associated with infection from Epstein-Barr virus (EBV), which may primarily occur in the lungs, thymus, gastrointestinal tract and urinary bladder as well [1]. LELC of the urinary bladder is a rare histological type of malignant tumour, representing 0.4%-1.3% of all bladder cancers [2]. Three main types of LELC have been described according to the proportion of lymphoepithelial tissue component within the tumour: pure (100%), predominant (50%-99%) and focal (<50%) [3], with the better prognosis being reported for the pure and predominant types as compared to the focal type [4].

No standard treatment is followed for patients presenting with LELC of the urinary bladder, mainly due to the rarity of this malignancy and the variety of baseline patient profiles. Radical cystectomy followed by chemotherapy and transurethral resection of the tumour with chemotherapy and/or radiotherapy has been shown to be effective [5-8]. Most cases described in the literature have been described in patients <90 years old, with only one case reported in a 97-year-old woman who was treated with transurethral resection of the tumour without chemotherapy and no follow-up [9]. We present a case of LELC of the urinary bladder in a 93-year-old female patient who was treated with transurethral resection and cisplatin-based chemotherapy.

## Case presentation

**Figure 1:**
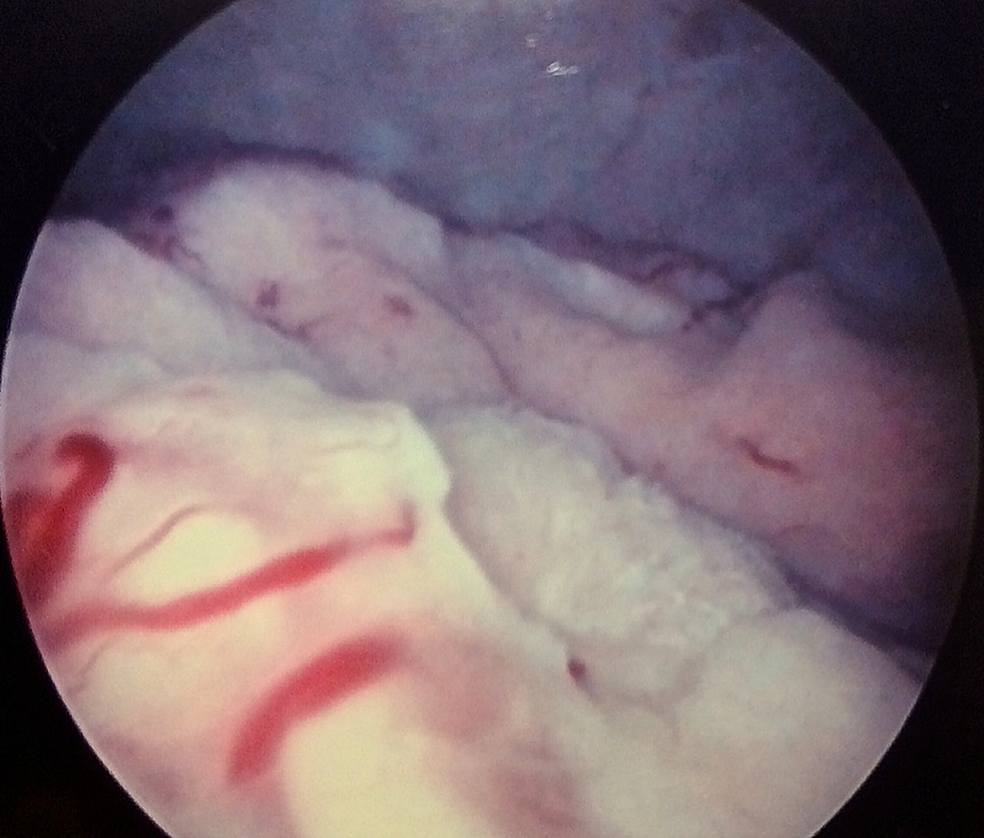
Cystoscopy view of a broad-based tumour protruding from the right bladder wall

A 93-year-old female patient was admitted to our department with gross haematuria and irritative symptoms during urination. She had a medical history of bladder tumour resection in the past with no information about the histology, while she suffered also from hypertension, diabetes mellitus and ischemic heart disease. Ultrasound scan, computed tomography and flexible cystoscopy disclosed a broad-based tumour located on the right bladder wall, with no associated hydronephrosis or abdominal/pelvic lymphadenopathy, which was subsequently resected under spinal anaesthesia (Figure 1). Urine cytology returned negative for abnormal findings.

**Figure 2:**
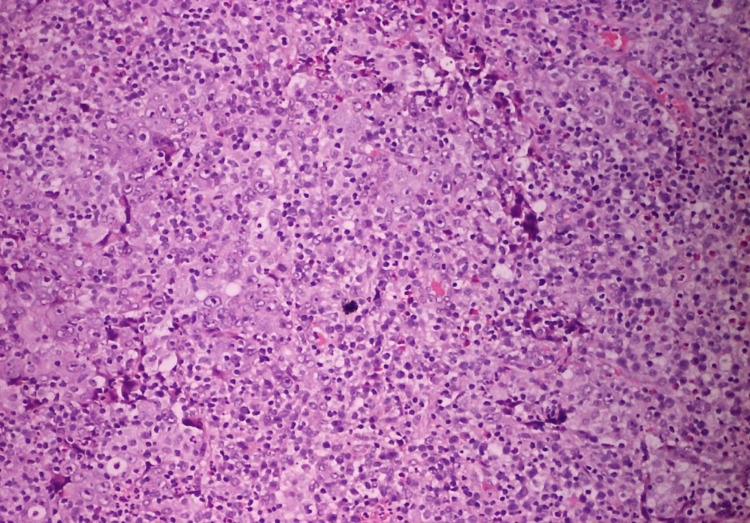
Sheets and cords of undifferentiated malignant cells arranged in syncytia, with frequent mitotic figures and a prominent chronic inflammatory infiltrate in the stroma, which is largely composed of lymphocytes, neutrophils, eosinophils and a few plasma cells

**Figure 3:**
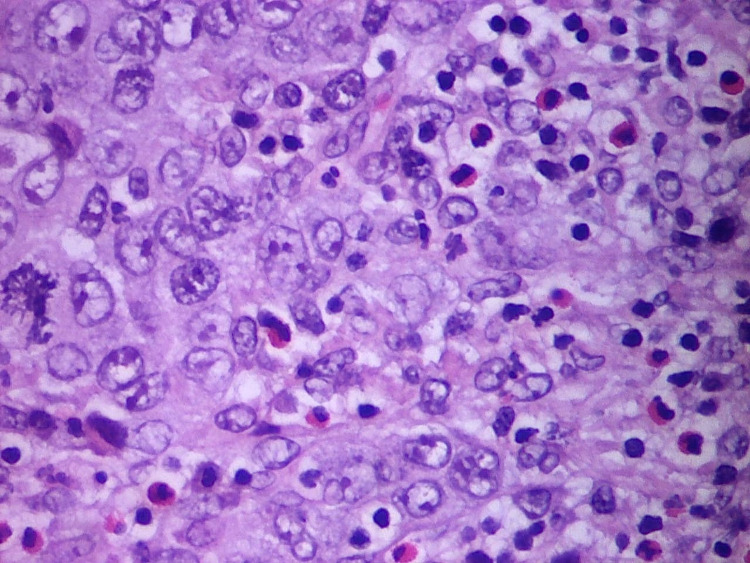
Higher magnification showing enlarged vesicular nuclei, prominent nucleoli, indistinct cell borders, mitotic figures and the inflammatory component

Histological specimens were prepared using formalin fixation and were stained with haematoxylin-eosin. Histological analysis revealed a high-grade tumour comprising of malignant cells forming sheets in a solid-syncytial pattern with intense nuclear atypia and prominent nucleoli (Figures 2, 3). There was an abundant inflammatory infiltrate comprised mainly of lymphocytes, both B- and T-cells, plasma cells and a small proportion of neutrophils. Immunohistochemical study revealed positivity for CD20 and CD3 for B- and T-cells, respectively, within the inflammatory infiltrate, while epithelial cells of the tumour stained positive for p63 and GATA3, but staining for chromogranin was negative. The tumour was found to invade muscularis propria (T2).

The patient, after an uneventful postoperative period, was referred to the Medical Oncology Department, where she was commenced on cisplatin-based chemotherapy, considering her age, comorbidities and performance status. Unfortunately, three weeks after initiation of chemotherapy the patient died of neutropenia-induced sepsis, most likely associated with chemotherapy.

## Discussion

Lymphoepitheliomas are undifferentiated malignant epithelial tumours of the nasopharynx characterised by a prominent lymphoid infiltrate [10]. LELCs demonstrate similar histological features but arise outside the nasopharynx [10]. The occurrence of the LELCs in the urinary tract is very rare with the bladder being the most common site [6,11]. Bladder LELCs were firstly described in 1991 by Zukerberg et al. [12] and represent 0.4%-1.3% of all bladder carcinomas showing a male predominance (70.9%) [2]. Up to date, 151 cases of LELC of the urinary bladder have been described, with most of the reports referring to case reports and case series of fewer than 5 patients, emphasizing the rare occurrence of this tumour [13]. Due to scarce descriptions of cases, no uniform management algorithms exist, with both radical cystectomy and more conservative treatments being reported [13].

Infection from EBV has been shown to be associated with the pathogenesis of LELCs in several tissues but not in the urinary bladder [4]. In contrast, p53 abnormalities seem to be crucial for the development of LELC of the urinary tract [10]. In their study in 34 patients, Williamson et al found that bladder LELCs share a number of features with conventional high-grade invasive urothelial carcinoma, including the frequent presence of urothelial carcinoma in situ (CIS), expression of p53 and p63 and molecular abnormalities detected by UroVysion fluorescence in situ hybridisation (FISH), suggesting similar pathogenesis of these two neoplasms [14].

Histologically, bladder LELCs are classified according to the amount of lymphoepithelial tissue within the tumour, into pure (100%), predominant (50%-99%) and focal (<50%), admixed with typical urothelial carcinoma, squamous cell carcinoma or adenocarcinoma [3]. Pathological features of the tumour include nests, sheets and cords of undifferentiated malignant cells, arranged in syncytia with ill-defined cytoplasmic borders and exhibiting vesicular, large nuclei, prominent nucleoli and frequent mitotic figures [14]. Glandular or squamous differentiation, as well as urothelial dysplasia or CIS, may be encountered [6]. Due to the prominent lymphoid background, composed of T and B lymphocytes, plasma cells, histiocytes and neutrophils or eosinophils, LELC of the bladder may be misdiagnosed as either reactive inflammatory lesion or lymphoma [3,6]. Immunohistochemistry is essential for the differential diagnosis and usually reveals positivity of tumour cells for various broad-spectrum epithelial markers, such as GATA3, epithelial membrane antigen, AE1/AE3, CK7 and CK8; in contrast, CK20 staining is frequently negative [14]. A diagnostic algorithm should also include imaging with a CT scan, ideally containing intravenous contrast to assess the upper urinary tract. Tumours are shown as filling gaps within the urinary bladder, commonly with a broader base compared to transitional urothelial carcinomas. Assessment of concurrent hydronephrosis, lymphadenopathy and metastatic disease should be performed also.

Due to the rarity of the disease, homogenous treatment strategies are lacking. Following initial transurethral resection of the tumour, radical cystectomy, radiotherapy and/or chemotherapy may be applied [4,14]. In the present case, surgery was not an option due to the patient’s advanced age and personal preferences. LELCs generally have a more favourable prognosis than conventional urothelial carcinomas of the bladder; also, the prognosis has been reported to be significantly better for the pure/predominant as compared to the focal type [14,15]. Nonetheless, Tamas et al. found no difference in the prognosis between pure and mixed LELCs treated by cystectomy, reporting a five-year recurrence-free risk of 59% (62% and 57%, for pure and mixed LELCs, respectively) [6]. Although the differences in the prognosis have not been well investigated, it has been postulated that the presence of lymph cells within the tumour implies an intense immune response, which apparently plays an important role in this regard [3].

In the present case, the clinical course was unfavourable and the patient died of neutropenia-induced sepsis, three weeks after initiation of chemotherapy.

## Conclusions

LELC of the urinary bladder is a rare clinical entity with undefined therapeutic strategies, which should be taken into consideration by clinicians in the case of patients presenting with bladder tumours. This case presented a woman of advanced age diagnosed with LELC of the bladder, who died from complications of chemotherapy after refusing to undergo radical cystectomy. Although either radical surgery or chemo/radiotherapy following initial transurethral excision of the tumour are indicated, clinicians should take into consideration patient comorbidities and performance status before the final decision.
